# Early Remdesivir for Patients Who Are Immunocompromised and Hospitalized With COVID-19

**DOI:** 10.1093/ofid/ofag630

**Published:** 2026-11-04

**Authors:** Kathleen E Hurwitz, Mark Berry, EunYoung Lee, Kayla Hendrickson, Nuvan Rathnayaka, Valentina Shvachko, Mazin Abdelghany, Carrie M Nielson, M Alan Brookhart, Anand Chokkalingam, Joshua A Hill

**Affiliations:** Epidemiology, Headwater Science, Durham, North Carolina, USA; Virology, Gilead Sciences, Foster City, California, USA; Virology, Gilead Sciences, Foster City, California, USA; Epidemiology, Headwater Science, Durham, North Carolina, USA; Epidemiology, Headwater Science, Durham, North Carolina, USA; Virology, Gilead Sciences, Foster City, California, USA; Virology, Gilead Sciences, Foster City, California, USA; Virology, Gilead Sciences, Foster City, California, USA; Department of Population Health Sciences, Duke University, Durham, North Carolina, USA; Virology, Gilead Sciences, Foster City, California, USA; Vaccine and Infectious Disease Division, Fred Hutchinson Cancer Center, Seattle, Washington, USA; Division of Allergy and Infectious Diseases, Department of Medicine, University of Washington, Seattle, Washington, USA

**Keywords:** clone-censor-weighting, COVID-19, hospitalization, immunocompromised, remdesivir

## Abstract

**Background:**

Patients who were immunocompromised were largely excluded from COVID-19 clinical trials, and real-world evidence on remdesivir effectiveness has produced conflicting results. We evaluated whether early remdesivir initiation (vs late or never) reduces in-hospital mortality among patients who are immunocompromised and hospitalized with COVID-19.

**Methods:**

We conducted a retrospective cohort study using a target trial emulation framework with clone-censor-weighting and deidentified hospital chargemaster data combined with medical and pharmacy claims from approximately 400 US hospitals. Patients included those who were immunocompromised, aged ≥12 years, and hospitalized for COVID-19 between 1 May 2020 and 3 December 2023. We assessed all-cause in-hospital mortality within 14 and 28 days of admission for 3 remdesivir treatment regimens: initiation within 2 days of admission (“early”), initiation within 3 to 7 days (“late”), and no initiation of remdesivir (“never”).

**Results:**

Among 97 587 eligible patients (median age, 71 years; 51% female), the 14-day cumulative risk of in-hospital mortality was 9.2% for early remdesivir, 10.1% for late, and 11.8% for never. At 14 days, the adjusted relative risk was 0.91 (95% CI, .86–.97) for early vs late initiation and 0.78 (95% CI, .75–.81) for early vs never initiation. At 28 days, trends were similar. Subgroup analyses by age, variant era, and immunosuppression severity yielded similarly protective effects of early initiation of remdesivir.

**Conclusions:**

The risk of in-hospital mortality for patients with COVID-19 who were immunocompromised was lower when remdesivir was administered early as compared with late or never. This result was consistent regardless of age, severity of immunosuppression, and variant era.

The COVID-19 pandemic was a global health event marked by nearly 6.5 million hospitalizations and 1.2 million deaths in the United States alone [1]. Infection with severe acute respiratory syndrome coronavirus 2 (SARS-CoV-2), the virus that causes COVID-19, is associated with flu-like symptoms, and in severe cases, patients develop lower respiratory infections and pneumonia symptoms that require hospitalization [1–3]. Patients with immunocompromising conditions are at increased risk of infection, severe disease, and mortality from COVID-19 [4–7]. In May 2020, remdesivir was the first therapy to be granted emergency use authorization by the Food and Drug Administration (FDA) for the treatment of COVID-19 [8]. On 22 October 2020, remdesivir was fully approved for the treatment of COVID-19 infection in hospitalized adults and pediatric patients (age ≥12 years) [9].

Patients who were immunocompromised were largely excluded from the early COVID-19 clinical trials [10, 11]. A previous observational study of such patients hospitalized with COVID-19 reported a significant reduction in inpatient mortality associated with initiation of remdesivir within 2 days of hospitalization (vs no remdesivir) [12]. While these results are encouraging, the clinical benefit of initiating remdesivir later in the hospitalization was not described. Additionally, understanding how the effectiveness of remdesivir differs by patient characteristics such as age, COVID-19 variant, and degree of immunosuppression would support decision making in the hospital setting. Finally, the prior study's use of propensity score matching—a common approach for comparative effectiveness analyses of real-world data—may inadequately control for clinical characteristics that evolve over time and affect the use and timing of remdesivir [13].

We sought to evaluate the effectiveness of early initiation of remdesivir (vs late or never) for preventing in-hospital mortality among immunocompromised cases of COVID-19 using a novel methodological approach that adjusts for time-varying confounding without introducing immortal time bias (ie, bias that can occur when the start of follow-up does not align with treatment assignment). We also conducted subgroup analyses to examine any variation in the effectiveness of early initiation of remdesivir by age group, variant era, or severity of immunosuppression.

## METHODS

### Study Design and Population

We conducted a retrospective observational cohort study using deidentified hospital chargemaster data combined with medical and pharmacy claims from approximately 400 hospitals across the United States from the HealthVerity data ecosystem [14–16]. Patients aged ≥12 years with a qualifying immunosuppressive condition who were hospitalized with COVID-19 between 1 May 2020 and 3 December 2023 were eligible for inclusion. Pediatric patients were included to match FDA-approved indication for remdesivir. Immunosuppressive conditions were identified through combinations of diagnosis codes (*ICD-10-CM*), procedure codes (*ICD-10 Procedure Coding System*, *CPT*, *Healthcare Common Procedure Coding System*), and medication codes (*National Drug Code*). Codes included the following: HIV/AIDS, hematologic malignancy with evidence of treatment within 180 days of hospitalization, other immune conditions (eg, neutropenia, myelofibrosis, leukocyte diseases), solid malignancy with evidence of treatment within 365 days of hospitalization, organ transplant with evidence of immunosuppressive medication within 365 days of hospitalization, and rheumatologic/inflammatory conditions (eg, multiple sclerosis, lupus erythematosus, rheumatoid arthritis). Severity was defined by National Institutes of Health criteria for degree of immunosuppression, which distinguish patients on the basis of current or recent immunosuppressive treatment intensity rather than the qualifying condition alone [17]. For example, to qualify as severe/moderate, active treatment for patients with solid tumor or hematologic malignancy may be an antineoplastic drug claim or chemotherapy administration code within 180 days of hospitalization; in contrast, a patient with solid malignancy diagnosed and treated years earlier with no current evidence of antineoplastic therapy was classified as having a mild immunosuppressive condition. The supplementary materials provide the definitions and code lists used to identify the immunosuppressive conditions and categorize the severity as mild or moderate/severe at the time of hospitalization. Last, to ensure accurate assessment of comorbidities, we required patients to have activity in the database (defined as ≥1 medical claim or chargemaster record) ≥12 months prior to hospitalization.

We excluded patients who were hospitalized for COVID-19 or received remdesivir at any time prior to the emergency use authorization for remdesivir or in the 90 days prior to the qualifying hospitalization. Patients were also excluded if they were pregnant or participated in a clinical trial during the 12 months prior to hospitalization; had an emergency department observational unit stay ≥2 days prior to first date of hospitalization; transferred from another hospital, hospice, or unknown location prior to admission; or were missing data on age, sex, or geographic region. Among patients with multiple eligible hospitalizations during the study period, we selected the earliest.

### Treatment Patterns

We visualized treatment patterns across 28 days of hospitalization using Sankey diagrams [18]. Sankey diagrams depict the flow of patients between states of disease treatment at predetermined timepoints. At each timepoint of interest (days 1, 2, 7, 14, and 28), we calculated the cross-sectional prevalence of the following states: remdesivir alone, dexamethasone alone, remdesivir plus dexamethasone, other treatments (eg, COVID-19–related treatments other than remdesivir or dexamethasone, such as oral antivirals, convalescent plasma, and biologic immunomodulators), no treatment, death, and discharge from hospital.

### Exposure

We assessed 3 treatment regimens defined according to use and timing of remdesivir: early initiation of remdesivir, defined as initiation on the day of or day following hospital admission (ie, day 1 or 2); late initiation of remdesivir, defined as initiation between days 3 and 7 (inclusive) of hospital admission; and never initiation of remdesivir, defined as no initiation during hospital admission. Remdesivir administration was identified by vendor charge codes, keyword search, and/or procedure codes (supplementary materials). The prespecified treatment regimen comparisons included early vs late initiation of remdesivir and early vs never initiation of remdesivir.

### Outcomes

Patients were indexed on the day of admission and followed for up to 28 days. The primary outcome was all-cause in-hospital mortality. We also evaluated a secondary composite outcome of all-cause in-hospital mortality or initiation of mechanical ventilation (MV) or extracorporeal membrane oxygenation (ECMO), whichever occurred first. The cumulative incidence of each outcome was measured at 14 and 28 days. In-hospital mortality was identified through discharge dispositions of “expired” or “discharged to hospice.” For patients with an “expired” disposition, date of death was set to the discharge date; for patients discharged to hospice, date of death was set to length of stay plus 90 days (eg, midpoint for life expectancy after discharge to hospice).

### Covariates

Demographics (age, sex, geographic region, insurance type, month/year of hospitalization) and hospital characteristics (number of beds, urban/rural location, academic/community) were assessed at hospital admission. A total of 16 comorbidities were considered according to medical conditions identified by the Centers for Disease Control and Prevention as risk factors for severe illness or death from COVID-19 [19]. Comorbidities were identified during the 365 days prior to hospital admission using any medical claim from an inpatient or outpatient encounter with the associated *ICD-10-CM* diagnosis code (supplementary materials). Persons who did not meet the definition of a medical condition were assumed to not have the condition [20].

Time-varying covariates included oxygen support (none, low flow, high flow, MV/ECMO) and use of other COVID-19 treatments, including oral antivirals, dexamethasone, biologic immunomodulators, antibody-based therapies, and other therapies (eg, anticoagulants, vasopressors). Drug administration and oxygen support were identified by vendor charge codes (supplementary materials).

### Statistical Analysis

We estimated the 14- and 28-day cumulative incidence of the primary and secondary outcomes using a target trial emulation with a clone-censor-weight design [21]. This approach reduces bias due to time-varying confounding influenced by past treatment, informative censoring, and immortal person-time, allowing for estimation of risk when treatment may depend on patient characteristics that evolve with time (supplementary materials) [22]. The study was designed to target the same parameter (ie, average treatment effect) that would be obtained in a hypothetical target trial in which all members of the study population were randomized to the treatment regimen of interest. For each of the 3 treatment regimens, the entire cohort was cloned. Within each copy, patients were followed until discharge, until death (or for the secondary outcome, initiation of MV or ECMO), or until they were nonadherent to the treatment regimen under study, whichever came first. On each day of follow-up, patients were weighted by the inverse of the probability of remaining adherent to the treatment regimen under study, conditional on a set of baseline and time-varying confounders [23]. We estimated the censoring probabilities by fitting a Cox proportional hazards regression model with the following covariates: age at admission, geographic region of hospital, history of disability at admission, time-updated oxygen support, time-updated dexamethasone use, and time-updated interactions between oxygen support and dexamethasone [16]. Initial fits of the censoring model yielded extreme weights for a minority of patients, which resulted in unstable effect estimates. To reduce the variance of the weighted effect estimates, we applied a 0.5% symmetric trimming of the study population, in which patients with weights at or beyond the 0.05 and 99.5 percentiles were removed [24].

A weighted Aalen-Johansen estimator was used to obtain separate estimates of the adjusted 14- and 28-day cumulative risks, adjusted risk differences, and adjusted risk ratios (aRRs) for the primary and secondary outcomes [25]. Separate analyses were undertaken for each of the 2 prespecified treatment regimen comparisons. We constructed 95% CIs using a nonparametric cluster bootstrapping procedure to account for the correlation between cloned copies of the cohort [26]. All analyses were conducted in R version 4.3.0 [27].

### Subgroup and Sensitivity Analyses

We performed subgroup analyses to assess variation in the effectiveness of the remdesivir treatment regimens by age category (12–64, ≥65 years), severity of immunosuppression (mild, moderate/severe), and calendar time defined by emergence of COVID-19 variants: pre-Delta (1 May 2020–31 May 2021), Delta (1 June 2021–30 November 2021), and Omicron (1 December 2021–end of data). We conducted a test of homogeneity of the subgroup-specific risk differences and risk ratios using the Cochrane *Q* test [28]. That is, we estimated the subgroup-specific risk differences and risk ratios for each subgroup and then compared the subgroup-specific effect estimates with the risk difference and risk ratio estimated from the overall study sample. All analyses were prespecified prior to receipt of the data; after completion of the overall analyses, we updated our precision estimates with the observed sample sizes for each treatment regimen before proceeding with the subgroup analyses. Considering the updated precision calculations, we did not conduct subgroup analyses for contrasts involving late initiation of remdesivir.

Last, the overall analysis included those patients hospitalized with COVID-19 as the primary admitting diagnosis. We conducted a sensitivity analysis in which we repeated the overall analyses among patients with a COVID-19–admitting diagnosis in any position (ie, COVID-19 did not have to be the primary reason for admission), resulting in a more generalizable patient cohort.

## RESULTS

### Study Population

We identified 246 889 patients who were immunocompromised and hospitalized with COVID-19 between 1 May 2020 and 3 December 2023 (Supplementary Figure 1). A total of 154 850 (63%) met eligibility criteria. Among the 154 850 patients with a COVID-19 diagnosis, 97 587 (63%) had COVID-19 as the primary admitting diagnosis and were included in the analysis. A total of 53 471 (55% of 97 587) patients never initiated remdesivir. Among the 44 116 who initiated remdesivir, 39 315 (89%) initiated remdesivir early (day of or day following admission), and 4432 (5%) initiated remdesivir late (between days 3 and 7).

Table 1 summarizes the demographic and clinical characteristics of the study population on the day of hospital admission, overall and by use and timing of remdesivir. Overall, two-thirds of the cohort was aged ≥65 years, with an even division between females (51%) and males (49%). Approximately 44% of qualifying hospitalizations occurred during the pre-Delta era, followed by the Omicron (40%) and Delta (15%) eras. Most patients (83%) had a mild immunocompromising condition, the most prevalent being solid malignancy (61%), followed by a rheumatologic or inflammatory condition (44%). Upon admission, most patients (73%) did not require oxygen support on hospital day 1. Among patients who initiated remdesivir at any time, the proportion who initiated early was comparable across subgroups when stratified by age group, variant period, sex, geographic region, insurance type, hospital size, certain comorbidities, severity of immunosuppressive conditions, certain baseline medications, and baseline oxygen support.

**Table 1.** Baseline Demographic and Clinical Characteristics of Patients Immunocompromised and Hospitalized With a Primary Diagnosis of COVID-19 in the United States Between 1 May 2020 and 3 December 2023

	Use and Timing of Remdesivir During Hospitalization, No. (%)			
	Overall (N = 97 587)	Early: Day 1 or 2 (n = 39 315)	Late: Days 3–7 (n = 4432)	Never: No Use (n = 53 471)
Age group, y				
12–29	1156 (1)	403 (1)	30 (1)	719 (1)
30–49	7911 (8)	3124 (8)	275 (6)	4494 (8)
50–64	22 668 (23)	9700 (25)	846 (19)	12 053 (23)
65–74	25 522 (26)	10 706 (27)	1262 (28)	13 463 (25)
75–80	16 399 (17)	6604 (17)	860 (19)	8864 (17)
≥81	23 931 (25)	8778 (22)	1159 (26)	13 878 (26)
Sex				
Female	49 822 (51)	19 754 (50)	2152 (49)	27 737 (52)
Male	47 765 (49)	19 561 (50)	2280 (51)	25 734 (48)
Variant era				
Pre-Delta	43 363 (44)	16 246 (41)	2576 (58)	24 288 (45)
Delta	14 913 (15)	7701 (20)	600 (14)	6573 (12)
Omicron	39 311 (40)	15 368 (39)	1256 (28)	22 610 (42)
Calendar quarter				
2020				
Apr–Jun	3586 (4)	72 (<1)	78 (2)	3418 (6)
Jul–Sep	6912 (7)	999 (3)	447 (10)	5410 (10)
Oct–Dec	15 695 (16)	6977 (18)	1182 (27)	7441(14)
2021				
Jan–Mar	13 982 (14)	6765 (17)	718 (16)	6427 (12)
Apr–Jun	3723 (4)	1703 (4)	175 (4)	1832 (3)
Jul–Sep	10 043 (10)	5429 (14)	433 (10)	4152 (8)
Oct–Dec	8261 (8)	3713 (9)	302 (7)	4229 (8)
2022				
Jan–Mar	12 258 (13)	4663 (12)	429 (10)	7137 (13)
Apr–Jun	3314 (3)	1273 (3)	99 (2)	1938 (4)
Jul–Sep	5432 (6)	1919 (5)	152 (3)	3352 (6)
Oct–Dec	4682 (5)	1793 (5)	151(3)	2729 (5)
2023				
Jan–Mar	3732 (4)	1433 (4)	91 (2)	2202 (4)
Apr–Jun	1429 (1)	557 (1)	42 (1)	829 (2)
Jul–Sep	2226 (2)	980 (2)	59 (1)	1117 (2)
Oct–Dec	2312 (2)	1039 (3)	74 (2)	1198 (2)
Severity of immunocompromising conditions				
Mild	80 952 (83)	32 344 (82)	3657 (83)	44 650 (84)
Moderate/severe	16 635 (17)	6971 (18)	775 (17)	8821 (16)
Immunocompromising condition^a^				
HIV/AIDS	1538 (2)	610 (2)	75 (2)	852 (2)
Hematologic malignancy	4936 (5)	2113 (5)	288 (6)	2506 (5)
Other immune condition	10 424 (11)	3930 (10)	471 (11)	5987 (11)
Solid malignancy	59 720 (61)	24 506 (62)	2745 (62)	32 261 (60)
Organ transplant	5455 (6)	2213 (6)	294 (7)	2919 (5)
Rheumatologic or inflammatory condition	43 175 (44)	16 704 (42)	1921 (43)	24 370 (46)
Baseline medications				
Oral antivirals^b^	36 (<1)	2 (<1)	1 (<1)	33 (<1)
Dexamethasone	44 297 (45)	24 319 (62)	1796 (41)	18 068 (34)
Biologic immunomodulators	2155 (2)	1486 (4)	36 (1)	627 (1)
Antibody-based therapies	2916 (3)	2183 (6)	68 (2)	658 (1)
Other therapies^c^	33 813 (35)	12 601 (32)	1389 (31)	19 702 (37)
Baseline oxygen support				
No oxygen	71 552 (73)	27 403 (70)	3348 (76)	40 517 (76)
Low-flow oxygen	13 523 (14)	6323 (16)	658 (15)	6507 (12)
High-flow oxygen	8729 (9)	3973 (10)	289 (7)	4438 (8)
Mechanical ventilation or ECMO	3783 (4)	1616 (4)	137 (3)	2009 (4)

Abbreviation: ECMO, extracorporeal membrane oxygenation.

^a^Percentages may sum to >100% as patients presented at baseline with multiple eligible immunocompromising conditions.

^b^Oral antivirals include nirmatrelvir/ritonavir and molnupiravir.

^c^Other therapies include anticoagulants, vasopressors, and antiplatelets.

### Treatment Patterns

Figure 1 presents the observed treatment patterns at predetermined timepoints during hospitalization. On the day of admission, 12% of patients initiated remdesivir alone, 27% initiated dexamethasone alone, 15% initiated remdesivir and dexamethasone, and 39% received none of the COVID-19–related treatments that we examined. Among patients who initiated remdesivir alone on day 1, 57% were discharged by day 7. Similarly, among patients who received dexamethasone only on day 1, 52% were discharged by day 7, and among patients with remdesivir and dexamethasone on day 2, 58.7% had been discharged by day 7.

**Figure 1.**
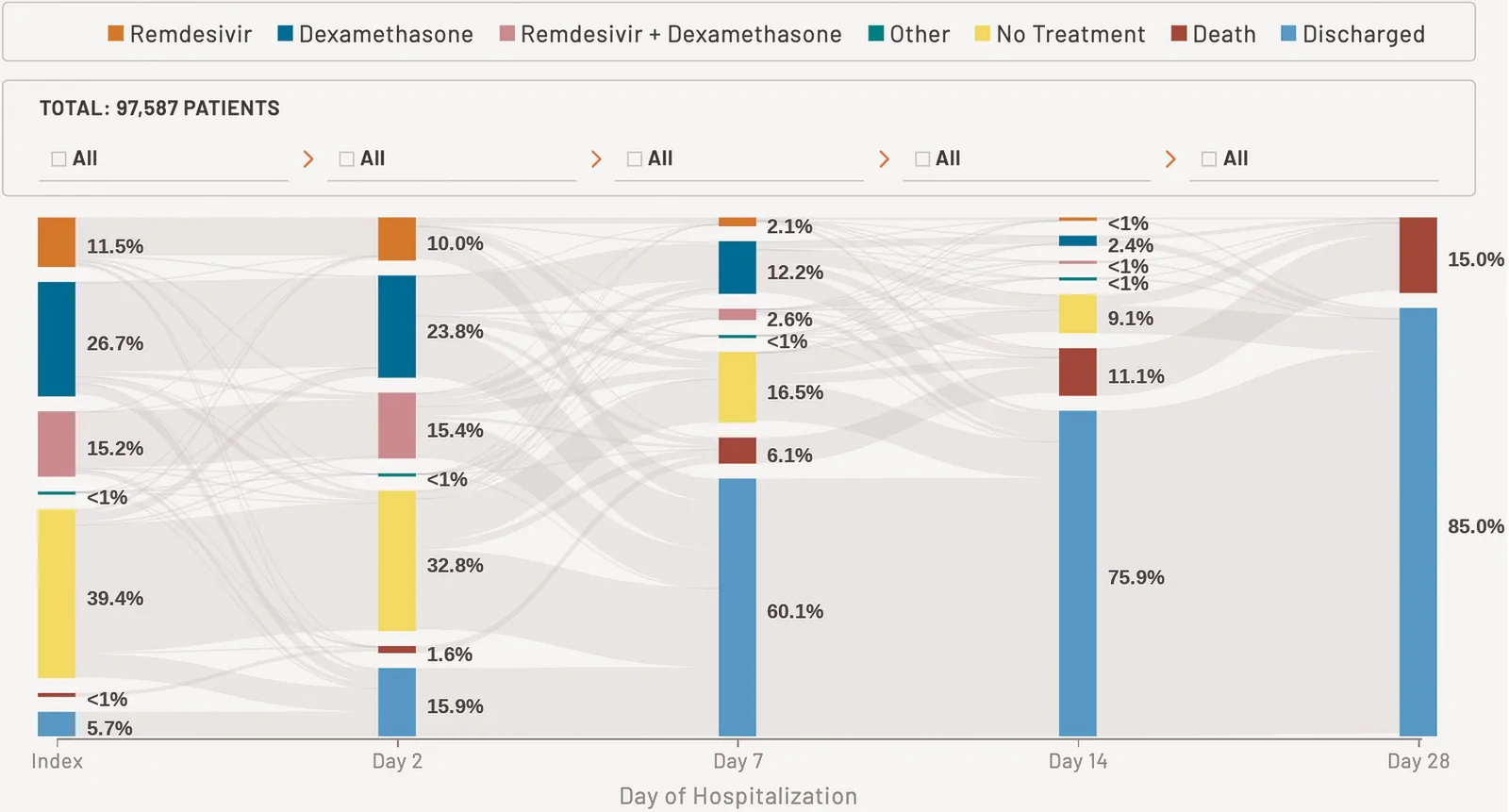
Treatment patterns over 28 days of hospitalization among patients who were immunocompromised and hospitalized with a primary diagnosis of COVID-19 in the United States between 1 May 2020 and 3 December 2023 (N = 97 587). Each vertical bar represents the proportion of patients in each treatment state at the indicated timepoint. Flows between bars represent transitions between treatment states. Treatment states include remdesivir only, dexamethasone only, remdesivir and dexamethasone combined, other treatment, no treatment, in-hospital death, and hospital discharge.

### In-Hospital Mortality

Figure 2 presents the adjusted cumulative incidence of in-hospital mortality assessed at 14 and 28 days after admission by treatment regimen. After adjustment and 0.5% symmetric trimming, the 14-day cumulative risk of in-hospital mortality was 9.2% (95% CI, 8.9%–9.5%) for the early initiation of remdesivir, 10.1% (95% CI, 9.6%–10.6%) for late initiation, and 11.8% (95% CI, 11.5%–12.2%) for never initiation. Cumulative risk at 28 days was similar: patients initiating remdesivir early experienced the lowest risk of in-hospital mortality (13.7%; 95% CI, 13.3%–14.0%), followed by late (14.5%; 95% CI, 13.9%–15.3%) and never (16.6%; 95% CI, 16.2%–16.9%). Comparison of the remdesivir regimens at 14 days for early vs late and early vs never yielded aRRs of 0.91 (95% CI, .86–.97) and 0.78 (95% CI, .75–.81), respectively (Table 2). That is, early initiation was associated with a 9%–22% reduced risk of in-hospital mortality at 14 days. The aRRs for both treatment comparisons at 28 days were attenuated, relative to 14 days, but remained statistically significant. When we compared the treatment regimens on the absolute risk difference scale, the results were consistent.

**Figure 2.**
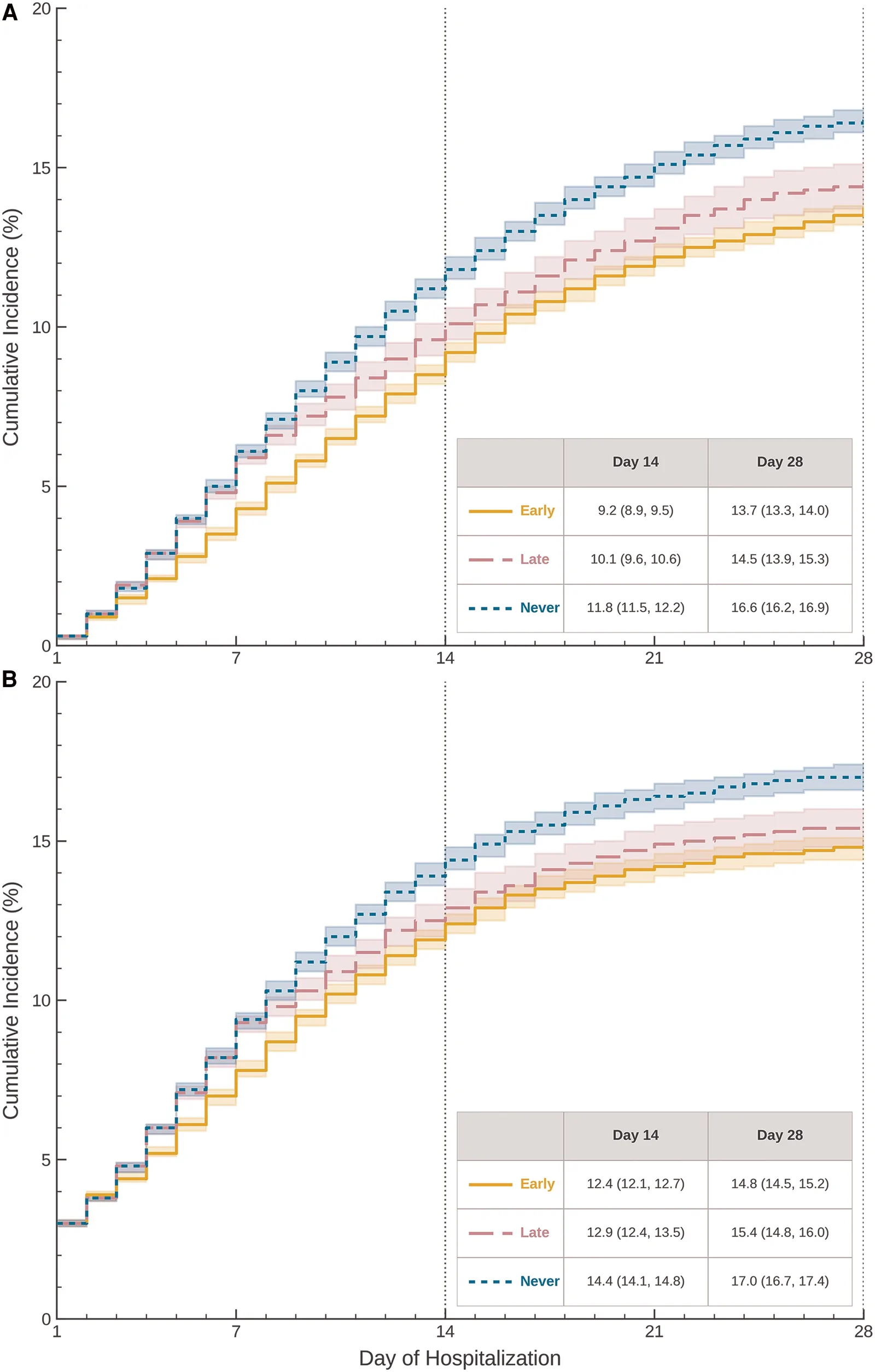
Adjusted cumulative incidence and 95% CIs (shaded bands) of (*A*) the primary endpoint of in-hospital mortality and (*B*) the secondary composite endpoint of in-hospital mortality, mechanical ventilation, or extracorporeal membrane oxygenation according to remdesivir treatment timing (solid line, early; long-dashed line, late; short-dashed line, never) among patients who were immunocompromised and hospitalized with a primary diagnosis of COVID-19 in the United States between 1 May 2020 and 3 December 2023. Inset tables show point estimates and 95% CIs at days 14 and 28. All analyses adjusted for age group, calendar quarter, geographic region, disabilities, time-varying oxygen support, time-varying use of dexamethasone, and interaction of time-varying oxygen support and dexamethasone use.

**Table 2.** Adjusted 14- and 28-Day Cumulative Risk Differences, Risk Ratios, and 95% CIs for In-Hospital Mortality and a Composite Endpoint of In-Hospital Mortality, MV, or ECMO

	aRD (95% CI), %^a^		aRR (95% CI)^a^	
Endpoint and Timing of Assessment	Early vs Late	Early vs Never	Early vs Late	Early vs Never
Primary: in-hospital mortality^b^				
Day 14 of hospitalization	−0.89 (−1.49, −.29)	−2.61 (−3.06, −2.17)	0.91 (.86, .97)	0.78 (.75, .81)
Day 28 of hospitalization	−0.88 (−1.66, −.10)	−2.90 (−3.39, −2.41)	0.94 (.89, .99)	0.82 (.80, .85)
Secondary: in-hospital mortality or initiation of MV or ECMO^b^				
Day 14 of hospitalization	−0.53 (−1.14, .08)	−2.03 (−2.49, −1.58)	0.96 (.91, 1.00)	0.86 (.83, .89)
Day 28 of hospitalization	−0.58 (−1.26, .09)	−2.21 (−2.69, −1.73)	0.96 (.92, 1.00)	0.87 (.84, .90)

Abbreviations: aRD, adjusted risk difference; aRR, adjusted risk ratio; ECMO, extracorporeal membrane oxygenation; MV, mechanical ventilation.

^a^Early remdesivir defined as initiation of remdesivir on the day of or day following hospital admission (ie, day 1 or 2); late remdesivir defined as initiation between days 3 and 7 (inclusive) of hospital admission; never remdesivir defined as no initiation of remdesivir during hospital admission.

^b^All analyses adjusted for age group, calendar quarter, geographic region, disabilities, time-varying oxygen support, time-varying use of dexamethasone, and interaction of time-varying oxygen support and dexamethasone use.

### In-Hospital Mortality, MV, or ECMO

The 14-day cumulative risks of the secondary composite outcome of MV/ECMO or in-hospital mortality were 12.4% (95% CI, 12.1%–12.7%), 12.9% (95% CI, 12.4%–13.5%), and 14.4% (95% CI, 14.1%–14.8%) for early, late, and never remdesivir, respectively (Figure 2). Cumulative risks at 28 days were similar. Comparison of the remdesivir regimens at 14 and 28 days for early vs late and early vs never yielded similar albeit attenuated, relative to the primary endpoint, aRRs and adjusted risk differences (Table 2).

### Subgroup Analyses: Age, Variant Era, and Immunosuppression Severity

Figure 3 summarizes the aRRs comparing the remdesivir regimens of early vs never initiation for the prespecified subgroup analyses of in-hospital mortality evaluated at 14 and 28 days. Supplementary Figure 2 provides corresponding subgroup results for in-hospital mortality evaluated on the risk difference scale at 14 and 28 days. Overall, we detected heterogeneity of the effect of early initiation of remdesivir by age (*P* = .02) and variant era (*P* = .04). This demonstrated that among patients aged 12 to 64 years, the effect of early (vs late) initiation of remdesivir on in-hospital mortality at 14 days was stronger (aRR, 0.70; 95% CI, .63–.77) than the effect estimated in the overall population (aRR, 0.78; 95% CI, .75–.81). Among patients aged ≥65 years, the estimated effect (aRR, 0.79; 95% CI, .75–.83) was similar to the results in the overall population. For the subgroup analysis by variant era, the effectiveness of early initiation of remdesivir among patients hospitalized during the Delta wave was weaker though still significant (aRR, 0.89; 95% CI, .83–.96) as compared with the overall treatment effect. Similar estimates of treatment effectiveness were observed among patients hospitalized during the pre-Delta (aRR, 0.80; 95% CI, .77–.84) and Omicron (aRR, 0.82; 95% CI, .77–.87) eras. There was no evidence of treatment effect heterogeneity by severity of immunosuppression at baseline (*P* = .18).

**Figure 3.**
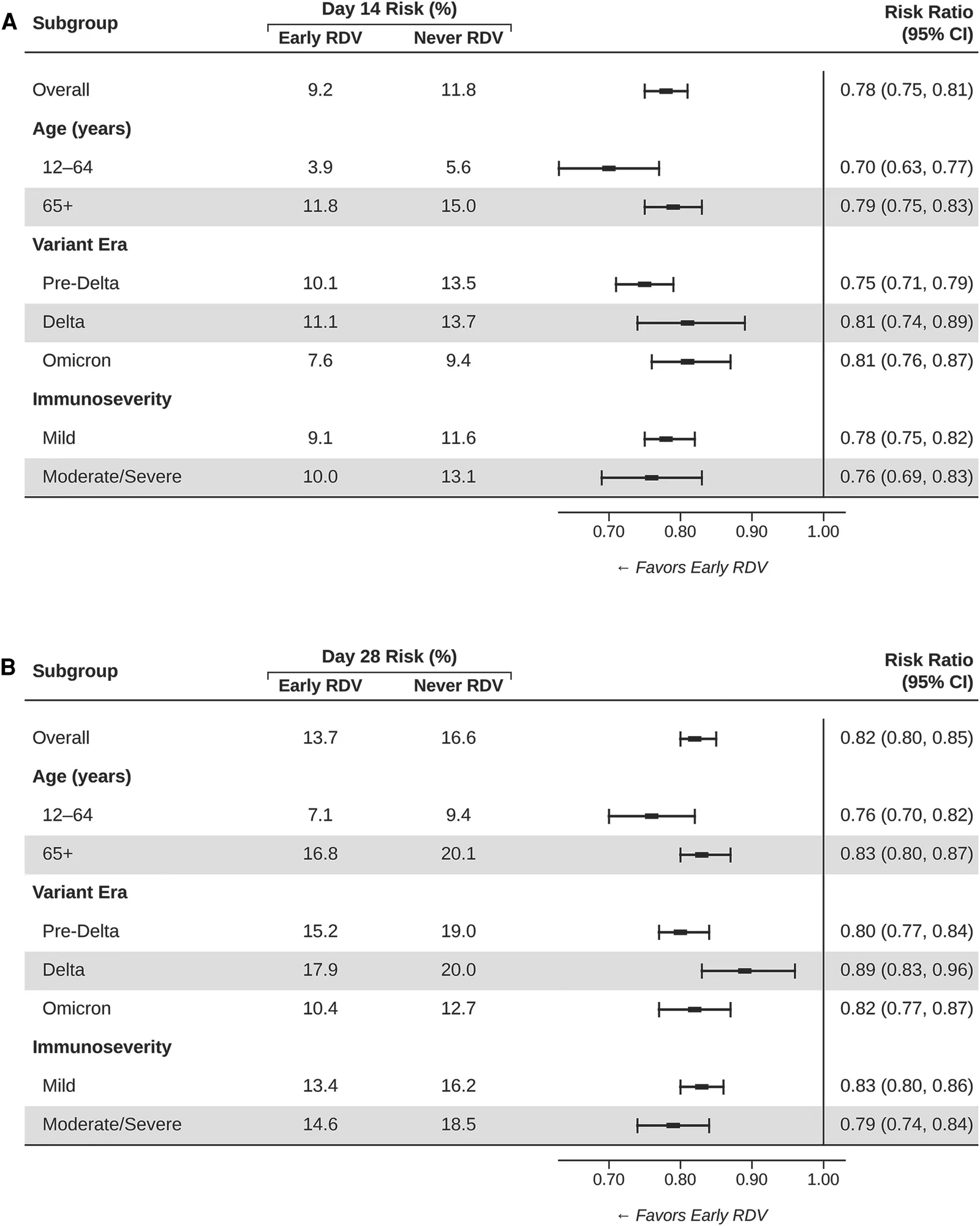
Adjusted subgroup analyses by age group, variant era, and severity of immunosuppression for (A) 14-day and (B) 28-day in-hospital mortality among patients who were immunocompromised and hospitalized with a primary diagnosis of COVID-19 in the United States between 1 May 2020 and 3 December 2023. RDV, remdesivir.

## DISCUSSION

This study examined the effectiveness of remdesivir use and timing among patients who were immunocompromised and hospitalized with COVID-19 to prevent in-hospital mortality. We found that initiating remdesivir on the day of admission or the following day conferred the greatest survival benefit, reducing the relative risk of 14-day in-hospital mortality by 22% as compared with never initiating remdesivir. Results were consistent, though attenuated, for the composite outcome of in-hospital mortality or receipt of MV/ECMO such that early initiation of remdesivir reduced the 14-day risk by 14%. Of note, nearly 55% of patients never initiated remdesivir during their hospitalization, even though it was FDA approved and guideline recommended during the study period, underscoring an opportunity to improve clinical outcomes by utilizing all available therapies. The results of our prespecified subgroup analyses demonstrated a robust, protective effect of remdesivir regardless of age group, severity of immunosuppression, and variant era. That is, across all subgroups, the aRR for in-hospital mortality evaluated at either 14 or 28 days showed a statistically significant survival benefit of early initiation of remdesivir.

This study had several strengths, the primary of which was the use of a rigorous analytic method that appropriately evaluates treatment decisions that evolve over time (and may not overlap with hospital admission) in response to time-varying patient characteristics. Additionally, the approach allowed us to study *all* eligible patients regardless of use and timing of remdesivir. That is, it explicitly targeted the same parameter that would have been obtained in a hypothetical target trial in which everyone was randomized to follow each of the 3 treatment regimens. Conventional designs such as propensity score matching, in which treated patients are matched to 1 or more untreated patients, do not generalize to the overall population. Instead, it estimates the impact of treatment in the group of patients who were treated and for whom acceptable matches can be identified (ie, data from treated and untreated patients without available matches are thrown away).

This study is also subject to limitations. First, we were unable to adjust for receipt and timing of COVID-19 vaccination. These missing data would likely affect the subgroup analyses for variant era, as vaccines were unavailable during the initial COVID-19 wave but were widespread by the Omicron era. Although rising vaccination rates may attenuate the added benefit of remdesivir, our results show a persistent reduction in mortality across variant eras, suggesting that remdesivir still confers survival benefit to vaccinated patients. To quantify the potential impact of this unmeasured confounding, we calculated the E-value for the primary 14-day early-vs-never comparison: an unmeasured confounder would need to be associated with early remdesivir initiation and mortality by a risk ratio of at least 1.88, beyond the measured covariates already adjusted for, to fully explain away the observed association (E-value of 1.77 for the confidence interval limit closest to the null) [29, 30]. While vaccination status is likely associated with mortality in patients who are immunocompromised, there is no clear mechanism by which it would also be differentially distributed according to remdesivir initiation timing, making it unlikely that vaccination confounding alone accounts for the full magnitude of the observed effect.

Second, a minority of patients in this analysis had severe immunocompromising conditions—the subgroup expected to benefit most from remdesivir due to lower vaccine-induced protection and increased risk of disease severity. Nonetheless, we found no evidence of effect modification by severity of immunosuppression (*P* = .18), suggesting that the protective effect of early remdesivir extends to patients receiving less intensive or more remote immunosuppressive treatment and addressing, in part, whether treatment recency or ongoing immunosuppressive management modifies this benefit. Third, data on timing of in-hospital treatments may be captured via disparate mechanisms (eg, medications vs oxygen therapy), leading to imprecise time ordering in the data source of treatments in relation to their actual administration. Finally, we were unable to conduct subgroup analyses evaluating early vs late initiation of remdesivir or subgroup analyses by baseline oxygen support category, due to insufficient sample size. For example, among patients hospitalized during the Delta variant era, only 600 patients initiated remdesivir late. Precision analyses indicated that given our a priori assumptions, the observed sample size was insufficient to distinguish between random variability and a null result. The study team thus decided not to pursue this analysis, leaving an important opportunity for future work.

In conclusion, using rigorous methodology, we find that remdesivir improves survival among patients who are immunocompromised and hospitalized with COVID-19 and that timing of remdesivir initiation affects effectiveness, providing a more nuanced understanding to inform real-world use of remdesivir.

## Supplementary Material

ofag630_Supplementary_Data

## Supplementary Data

Supplementary materials are available at *Open Forum Infectious Diseases* online. Consisting of data provided by the authors to benefit the reader, the posted materials are not copyedited and are the sole responsibility of the authors, so questions or comments should be addressed to the corresponding author.
