# Supplementary material for: Early Remdesivir for Patients Who Are Immunocompromised and Hospitalized With COVID-19
Source: Open Forum Infect Dis. 2026 Nov 4;13(11):ofag630. doi: 10.1093/ofid/ofag630 (PMC13732734; doi:10.1093/ofid/ofag630)
Supplement: Supplementary file 1 [file ofag630_supplementary_data.zip › Supplemental_FigureS1_FlowDiagram.pdf]

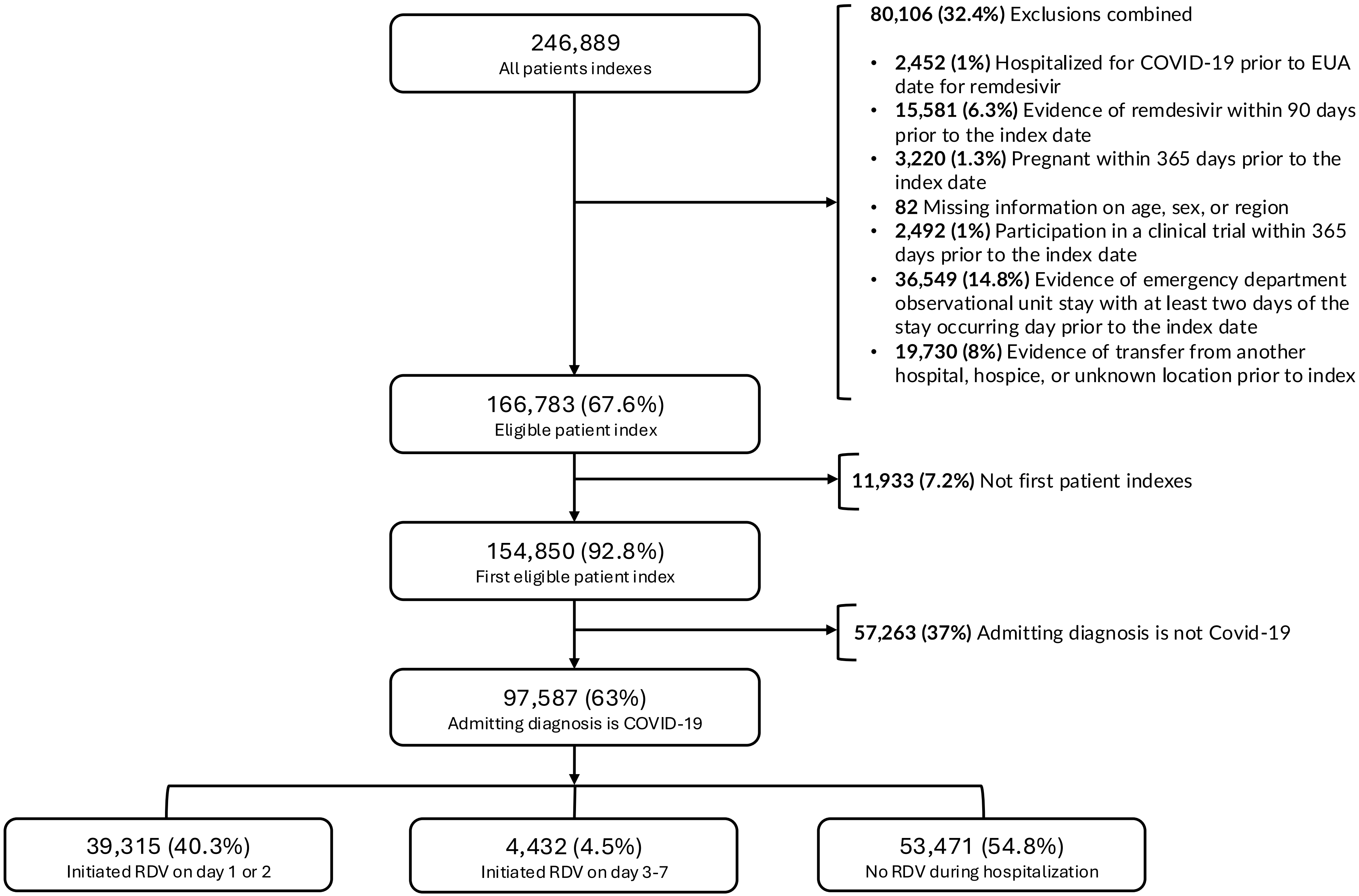
246,889
All patients indexes
80,106 (32.4%) Exclusions combined
2,452 (1%) Hospitalized for COVID-19 prior to EUA date for remdesivir
15,581 (6.3%) Evidence of remdesivir within 90 days prior to the index date
3,220 (1.3%) Pregnant within 365 days prior to the index date
82 Missing information on age, sex, or region
2,492 (1%) Participation in a clinical trial within 365 days prior to the index date
36,549 (14.8%) Evidence of emergency department observational unit stay with at least two days of the stay occurring day prior to the index date
19,730 (8%) Evidence of transfer from another hospital, hospice, or unknown location prior to index
166,783 (67.6%)
Eligible patient index
11,933 (7.2%) Not first patient indexes
154,850 (92.8%)
First eligible patient index
57,263 (37%) Admitting diagnosis is not Covid-19
97,587 (63%)
Admitting diagnosis is COVID-19
39,315 (40.3%)
Initiated RDV on day 1 or 2
4,432 (4.5%)
Initiated RDV on day 3-7
53,471 (54.8%)
No RDV during hospitalization
