# Supplementary material for: Early Remdesivir for Patients Who Are Immunocompromised and Hospitalized With COVID-19
Source: Open Forum Infect Dis. 2026 Nov 4;13(11):ofag630. doi: 10.1093/ofid/ofag630 (PMC13732734; doi:10.1093/ofid/ofag630)
Supplement: Supplementary file 1 [file ofag630_supplementary_data.zip › Supplemental_FigureS2_SubgroupAnalyses.docx]

**(a.)**

**(b.)**

**Supplemental Figures S2.a and S2.b** Adjusted risk differences (aRD) overall and by age group, variant era, and severity of immunosuppression for (a.) 14-day and (b.) 28-day in-hospital mortality among immunocompromised patients hospitalized with a primary diagnosis of COVID-19 in the United States between May 1, 2020, and December 3, 2023.
