# Supplementary material for: Early Remdesivir for Patients Who Are Immunocompromised and Hospitalized With COVID-19
Source: Open Forum Infect Dis. 2026 Nov 4;13(11):ofag630. doi: 10.1093/ofid/ofag630 (PMC13732734; doi:10.1093/ofid/ofag630)
Supplement: Supplementary file 1 [file ofag630_supplementary_data.zip › Supplemental_Methods.docx]

**Supplemental Methods**

To compare the effectiveness of the identified treatment regimens, we estimated the cumulative risk of each outcome using a target trial emulation approach based on the ‘clone-censor-weight’ design.^1^ This approach allows for the estimation of cumulative risks under dynamic treatment regimes in which treatment use may depend on patient characteristics that evolve with time. This reduces bias due to time-varying confounding impacted by past treatment, informative censoring, and immortal person-time.^1-3^ The study was designed to yield an estimate of the cumulative incidence of the outcome had, counter to fact, all individuals followed a particular treatment regimen. It explicitly targeted the same parameter that would be obtained in a hypothetical ‘target trial’ in which members of the study population were randomized to follow each of the treatment regimens.

Specifically, $Pr\left( Y\left( g \right)<t \right)$ was estimated with the following weighted estimator:

$\hat{Pr}\left( Y\left( g \right)<t \right)=\frac{1}{n}\sum_{i=1}^{n} \frac{I\left( T_{i,g}<t \right){I(J_{i,g} = 1)\Delta}_{i,g}}{\hat{Pr} \left( \Delta_{g}=1 | \bar{W}_{i}(T_{i,g}),T_{i,g} \right)}$	(1)

where $g$ was a treatment regimen of interest; $Y\left( g \right)$ was the time of the (possibly counterfactual) outcome that would be observed if a participant were treated according to protocol $g$; $T_{g}=min(Y,C_{g},D)$ was the earliest of the observed event time ($Y$), the time a participant was censored due to non-adherence to regimen $g$ ($C_{g}$), and the time the participant was censored for other reasons ($D$); $J_{g}$ takes value 1 when the event at time $T_{g}$ was the event of interest (and not a competing event); $\Delta_{g}=I(T_{g}=Y)$ was an indicator that the participant was uncensored at $T_{g}$; and  $\bar{W}(T_{g})$ was the history of the covariate process through $T_{g}$.

Effectively, the estimator reweighted the outcomes among the participants who remain uncensored under regimen $g$ to represent the full study cohort had all participants followed regimen $g$.

In practice, this estimator was implemented via the following algorithm:

1) Create one copy of each patient and their associated data for each treatment regimen
2) For each clone, a censoring time is computed corresponding to the time at which the patient stops following the treatment regimen corresponding to that copy of the data because of any of the following:
   a. Early remdesivir regimen: Participant will be censored if they do not initiate remdesivir on or before day 2
   b. Late remdesivir regimen: Participant will be censored if they initiate remdesivir on or before day 2 or if they do not initiate remdesivir between day 3 and day 7 (inclusive)
   c. No remdesivir regimen: Participant will be censored if they initiate remdesivir on any day during follow-up

The uncensored participants are then reweighted by the inverse of their probability of remaining uncensored conditional on a set of (baseline and time-varying) covariates sufficient to allow for sequential exchangeability.^4^

3) Fit a model for the probabilities of remaining uncensored using Cox proportional hazards regression and the Breslow estimator for the baseline hazard function.^5^ Covariates in the model will include both baseline and time-varying variables that together are designed to eliminate bias due to the informative censoring that occurs from regimen deviations. This model will be fit separately for each treatment regimen group, and treatment regimen nonadherence will be modelled separately from other sources of censoring.
4) Compute a weight for each uncensored index-copy at each time point of interest as the inverse of the probability of the patient remaining uncensored through that time point using the predicted probabilities obtained from the models fit at Step 3. Prior to applying the weights, we will examine their distribution (separately for each treatment regimen). If necessary, we will stabilize the weights to safeguard against model misspecification (e.g., extremely high or extremely low value for weights suggests key factors have either not been incorporated or incorporated incorrectly in the model for weights). In addition to stabilized weights, we will also consider truncating extreme weights by reassigning them to a threshold value.^6^ Within each copy of the data, the weights will allow those who followed a given treatment regimen to be weighted to represent the characteristics of both the censored and uncensored participants at each censoring time (e.g., through upweighting those individuals who were likely to be censored for regimen deviation, as a function of covariates, but instead adhered to a given protocol). After applying the weights, the uncensored population is expected have the same baseline and time-varying covariate distribution of the total study population had the entire population followed the treatment regimen of interest.
5) Estimate the counterfactual cumulative incidence of each outcome of interest using equation (1) above separately for each data copy.
6) Compute risk differences and risk ratios using the estimates from step 5.
7) Obtain confidence intervals using a nonparametric bootstrapping procedure in which participants are resampled with replacement a minimum of 300 times based on previous analyses so as to properly account for variance inflation due to the covariance between analytic units.^7^ However, additional bootstrap samples may be obtained if the empirical standard error fails to adequately converge based on standard criteria.^7-8^ For each resampled dataset, all aspects of estimation will be repeated, including the fitting of the treatment model, the construction of the weights, and the fitting of the weighted Cox proportional hazards model used to estimate the treatment effect. The 2.5 and 97.5 percentiles of the bootstrap distribution will be used to calculate the 95% confidence interval. Participants may contribute person-time to more than one treatment regimen, in the same or multiple regimen contrasts. Note that correct application of bootstrap sampling requires sampling at the patient level rather than the patient index level.

Three treatment regimens were analyzed using this approach. Each treatment regimen group included the same participants, meaning there were three copies of data with different censoring times for each treatment regimen.

The treatment regimens included:

1) Early remdesivir: Initiate remdesivir on or before day 2 of hospital admission
2) Late remdesivir: Initiate remdesivir between day 3 and day 7 (inclusive) of hospital admission
3) No remdesivir: Do not initiate remdesivir treatment during hospital admission

In **Regimen (1)** if participants did not receive remdesivir on or before day 2 or follow-up, they were censored at day three.

In **Regimen (2)** if participants received remdesivir on or before day 2 or follow-up, they were censored at that time. If participants did not receive remdesivir between day 3 and day 7 of follow-up, they were censored on day eight.

In **Regimen (3)** if participants received remdesivir at any time during follow-up, they were censored at that time.

The index date was the date of COVID-19-related hospital admission. Follow-up started on the first day (inclusive) following the index date and continued until the first of the following:

- date of outcome event
- date of noncompliance with the treatment regimen of interest
- end of follow-up (i.e., day 29 of follow-up)
- date of hospital discharge

Patients may have been discharged to different destinations, each of which affects how the end of follow-up was handled in the analysis. If the discharge destination implied that the patient was no longer at risk of the outcome, we treated that discharge destination as a competing event. For example, if a patient was discharged home, then they were no longer at risk for either in-hospital or mechanical ventilation or ECMO. Conversely, if the discharge destination did not preclude the outcome from occurring in-hospital, the discharge destination was considered a censoring event. For example, patients can be transferred to another hospital during their COVID-19 hospitalization, which would not prevent the patient from experiencing the outcome. The table below summarizes how each discharge destination was handled for each outcome.

Table. Discharge dispositions in the HealthVerity data and the analytic approach for end of follow-up for each outcome.

| - **Discharge disposition** | **Outcome** | |
| --- | --- | --- |
|  | **In-hospital mortality** | **In-hospital mortality or initiation of mechanical ventilation or ECMO** |
| Home | Competing risk | Competing risk |
| Skilled nursing facility or long-term care | Competing risk | Competing risk |
| Hospital transfer | Censoring | Censoring |
| Against medical advice | Censoring | Censoring |
| Hospice | Outcome | Outcome |
| Unknown | Censoring | Censoring |

**References**

1. Maringe C, Benitez Majano S, Exarchakou A, et al. Reflection on modern methods: trial emulation in the presence of immortal-time bias. Assessing the benefit of major surgery for elderly lung cancer patients using observational data. *International Journal of Epidemiology*. 2020;49(5):1719-1729. doi:10.1093/ije/dyaa057
2. Hernán MA, Lanoy, E., Costagliola, D., Robins, J.M. Comparison of dynamic treatment regimes via inverse probability weighting. *Basic & Clinical Pharmacology & Toxicology*. 2006;98(3):237-42.
3. Robins JM, Finkelstein, D.M. Correcting for noncompliance and dependent censoring in an AIDS clinical trial with inverse probability of censoring weighted (IPCW) log‐rank tests. *Biometrics*. 2000;56(3):779-88.
4. Robins J, Hernan, M. Estimation of the causal effects of time-varying exposures. *Handbooks of Modern Statistical Methods*. 2008:553-599.
5. Lin DY. On the Breslow estimator. *Lifetime Data Analysis*. 2007;13:471-80.
6. Lee BK, Lessler, J., Stuart, E. A. Weight trimming and propensity score weighting. *PloS One*. 2011;6(3):e18174.
7. Davison AC, Hinkley, D.V. Further ideas. *Bootstrap Methods and their Application*. Cambridge University Press; 2013:70-135.
8. North BV, Curtis, D., Sham, P.C. A note on the calculation of empirical P values from Monte Carlo procedures. *American Journal of Human Genetics*. 2002;71(2):439-41.
